# Nb and Cu co-doped (La,Sr)(Co,Fe)O_3_: a stable electrode for solid oxide cells

**DOI:** 10.1039/d0ra10313f

**Published:** 2021-03-11

**Authors:** D. M. Neacsa, K. Abbassi, H. Guesmi, P. L. Coddet, J. Vulliet, M. El Amrani, S. Dealmeida-Didry, S. Roger, V. Ta Phuoc, R. Sopracase, F. Gervais, C. Autret-Lambert

**Affiliations:** GREMAN, UMR7347 CNRS, Université de Tours, Parc de Grandmont F-37200 Tours France autret@univ-tours.fr; CEA DAM, Le Ripault F-37260 Monts France

## Abstract

Solid oxide cells (SOCs) are electrochemical devices that convert the chemical energy of a fuel into electricity. With regard to electrodes, the development of materials with mixed conduction properties is a key issue for improving the performance of SOCs at high temperatures. New Cu and Nb co-doping La_1−*x*_Sr_*x*_Fe_*y*_Co_1−*y*_O_3−*δ*_ (LSCF) materials were studied as electrode materials on yttria-stabilized zirconia (YSZ) supports. The results show that Cu_0.05_ + Nb_0.05_ co-doped LSCF maintains a stable cubic structure even after several heat treatments and has better conductivity than a classically used LSCF.

## Abstract 2

The stabilization of the cubic phase of LSCF co-substitution on the Fe site deposited on YSZ + GDC symmetrical cells to improve the performance.

## Graphical abstract

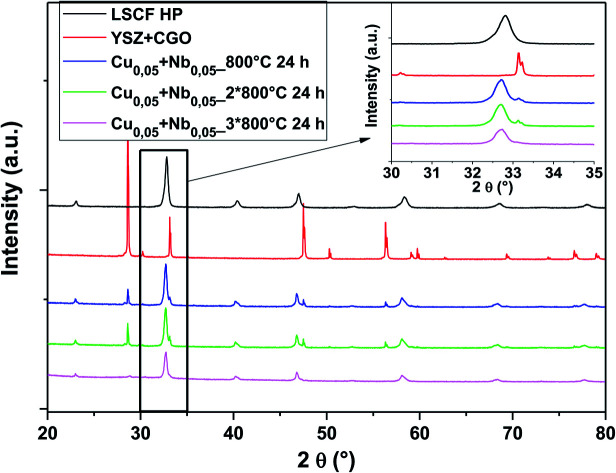

## 1. Introduction

Solid oxide cells (SOCs) appear to be a promising technology for the reversible conversion of gas to electricity.^1^ They are electrochemical devices that can reach high conversion efficiencies due to their high operating temperatures (800–1000 °C). Usually, hydrogen is used as a fuel and pure oxygen or oxygen air is used as an oxidizer. To increase their efficiency, particular attention must be paid to the development of electrode materials. Lanthanum strontium cobalt ferrite, La_1−*x*_Sr_*x*_Co_1−*y*_Fe_*y*_O_3−*δ*_ (LSCF), is one of the most developed oxygen electrodes used for SOCs. In recent years, this material has been intensively studied due to its better performance than the conventional La_1−*x*_Sr_*x*_MnO_3_ (LSM).^2^ In mixed ionic and electronic conductors (MIEC), the reaction can extend from the vicinity of triple phase boundaries (TPBs) to the whole surface area of the porous electrode, resulting in a substantial increase in performance. LSCF material crystallizes in an ABO_3_ perovskite structure in which A-sites are occupied by large La and Sr cations and B-sites are preferably filled by smaller Co and Fe cations. Different crystallized phases can be found for LSCF depending on the synthesis method and sintering temperature, such as an orthorhombic, rhombohedral or cubic structure.^3,4^

This compound exhibits high catalytic activity as well as good mixed electronic–ionic conductivity.^5–7^ The transition metals at B sites are responsible for the electrical conductivity.^7^ A site cations, especially the concentration of Sr at A-sites due to the replacement of La by Sr (cation with lower valence), induce the formation of oxygen vacancies and the appearance of ionic conductivity.^7–9^

The main issue for SOC development consists of degradation during operation at high temperature. Considering the LSCF material, main degradation mechanisms are due to the instability of the structure with temperature and the Sr segregation at the electrode/electrolyte interface.

To overcome the performance instability of LSCF, cubic structure is the privileged phase. It is considered to be the most stable structure with the other components of the cell, including the yttria-stabilized zirconia (YSZ) electrolyte.^10^ Agun^11^*et al.* reported that LSCF powder mixed with a conducting ceramic electrolyte material, namely samarium-doped ceria (SDC) – carbonate, overcomes both durability and stability issues. Data in the literature show that an evolution to rhombohedral phase is often observed^12^ and indicate that a careful study of the stability of LSCF materials with regard to synthesis conditions and substitutions is necessary.

Practical application of LSCF materials is also affected by their performance degradation due to Sr segregation and migration. The degradation appears first on the electrode/electrolyte interface, which creates resistive undesired Sr-phases.^13,14^ Then, Sr species, which are highly mobile, migrate through the layer to the electrolyte to form resistive SrZrO_3_ phases.^15^ The Sr segregation appears to originate from electrostatic interactions. Two main mechanisms have been identified to understand Sr surface segregation: the existence of internal strain because of the different sizes of the two occupants of A-sites^16,17^ and the electrostatic attraction of the Sr dopant in A-sites by oxygen vacancies due to the substitution of trivalent La^3+^ with bivalent Sr^2+^.^16,18^ One of the most reported solutions to suppress Sr surface segregation is doping of the B-sites with high valence cations such as Nb,^19,20^ Ce,^21^ Y,^22–24^ Ta,^25^ and Cu.^12,26^

Nb appears to be the best choice to enhance the chemical stability with doping of the B-sites because it is considered to be the element that copes best with the cubic structure in the LSCF network. Due to its ionic radius in the B-sites, Nb^5+^ (0.64 Å) creates more space for the large Sr^2+^ cations and more accommodation in the A-sites. Therefore, the Sr activation is reduced by expanding the LSCF lattice. Chen *et al.*^19^ showed that a small amount of Nb significantly enhances the stability of La_0.24_Sr_0.16_Ba_0.6_Co_0.5_Fe_0.44_Nb_0.06_O_3−*δ*_ electrode and inhibits poisoning with Cr from interconnections. However, it decreases the electrocatalytic activity of doped LSCF by reducing the oxygen reduction reaction (ORR).

Other researchers focused on two possibilities to improve the electrode performance: by metal surface modification^26^ or by B-site substitution in LSCF.^26^ Adding a third metal to the B-site seems to have an important impact on the properties of these materials. The addition of a small amount of Pd to the B-site enhances the properties of LSCF.^19^ An Nb and Ta co-substituted perovskite SrCo_0.8_Nb_0.1_Ta_0.1_O_3−*δ*_ as an electrode material was reported to exhibit structural stability and high electroactivity.^25^

Among all the elements, other well-known electrical conductor metals such as Pt, Pd, Ag and Cu can be used to improve the electrical performance. Compared to the others, the relatively low cost of copper is interesting. Addition of copper can enhance the dissociation of O_2_ and increase the electrochemical reaction.^12^ Cu^2+^ (0.73 Å) is used for doping at B-sites because of its ionic radii is close to those of Co^2+^ and Fe^3+^.^26^

It is important to note the influence of the synthesis process, especially the sintering temperature and substitution cation sizes compared to the B-site cation sizes in LSCF ABO_3_ perovskite. This work focuses on Nb and Cu co-doped LSCF in order to understand the strong dependence of the structure stability and conductivity evolution on the doped electrode after several heat treatments.

## 2. Experimental

The powders (standard LSCF (La_0.6_Sr_0.4_Co_0.2_Fe_0.8_O_3−*δ*_) and doped LSCF with Nb and/or Cu (La_0.6_Sr_0.4_Co_0.1_Nb_0.1_Fe_0.8_O_3−*δ*_, La_0.6_Sr_0.4_Co_0.2_Fe_0.7_Cu_0.1_O_3−*δ*_, La_0.6_Sr_0.4_Co_0.2_Fe_0.7_Cu_0.1−*x*_Nb_*x*_O_3−*δ*_ where *x* = 0.03; 0.05)) were synthesized by an organic gel-assisted citrate process.^27^ The samples were named LSCF Nb, LSCF Cu and LSCF Cu_0.1−*x*_ + Nb_*x*_ for simplification. First, triammonium citrate as a chelating agent was added to an aqueous solution of metal (M) nitrates with M = La, Sr, Co, Fe, Cu in stoichiometric amounts. Niobium acetate (C_4_H_4_NNbO_9_) was then added, and a clear solution that was stable up to gel pyrolysis was obtained. The gel was formed *in situ* with an auxiliary three-dimensional polymeric network. The monomers acrylamide and *N*,*N*′-methylenediacrylamide were dissolved and co-polymerised by heating to about 100 °C and adding azobisisobutyronitrile (AIBN) as a radical polymerization initiator. The resulting gel was calcined at 650 °C in air for 6 h to obtain a nanometric black powder. This powder was ground by ball milling in iso-propanol for 2 h in a three-dimensional stirrer (Bioengineering-Inversina) with a rotation speed of 8. After drying, the powder was characterized, and several heat treatments between 800 °C and 1200 °C were performed in order to determine the adequate sintering temperature to obtain single-phase compounds.

These synthesized powders were used as electrode materials on commercial YSZ disks (8 mol% Y_2_O_3_–ZrO_2_, 25 mm in diameter, Fuel Cells Materials). An ink was prepared with 60% ink vehicle (Fuel Cells Materials) and 40% pure LSCF or Cu and/or Nb-doped LSCF powder. The resulting ink was applied by screen printing on the commercial electrolyte YSZ previously covered by a 4 μm thick Gd_0.10_Ce_0.90_O_1.95_ (GDC, Fuel Cells Materials) barrier layer. The cells were dried after each layer deposition at 60–65 °C for 30 minutes. Once the layers were deposited, the whole cell was heat-treated at 1000 °C for 1 h in order to eliminate the solvent.

To examine the chemical compatibility between the electrode and electrolyte materials, several heat treatments were applied to the cells at 800 °C (the operating temperature of the cell is 800–1000 °C) for 24 h each time (the operating time must be as long as possible with a target greater than 5000 h).

On the other hand, in order to characterize the properties of these new LSCF-doped compounds compared to commercial powder (LSCF HP, Fuel Cells Materials), cold pressed pellets 1 mm in thickness and 13 mm in diameter were made from the powders using an uniaxial press followed by an isostatic press. The pellets were sintered in air between 800 °C and 1200 °C in order to obtain the adequate sintering temperature to afford cubic/rhombohedral single-phase compounds.

For all the samples (powders, pellets and cells), the room temperature X-ray patterns were obtained by using a Bruker D8 Advance diffractometer (*θ*–*θ*) with Cu-Kα radiation. The wavelength of the incident X-ray was about 1.54 Å. The powder patterns were measured in the angular range of 20° < 2*θ* < 80° in steps of 0.02°. The diffraction pattern analysis was performed by the Rietveld method using the FullProf program.^28^ A pseudo-Voigt function was chosen for the line shapes of the diffraction peaks.

The electron diffraction (ED) and high resolution images were obtained for the electrode materials using a JEOL 2100 F electron microscope operated at 200 kV and equipped with an energy dispersive spectroscopy analyzer. Samples for TEM were prepared by crushing the crystallites in ethanol, and the small flakes were deposited on a holey carbon film.

The microstructures of the powder, pellets and cells were observed using a MESU 1644 – HR JEOL scanning electron microscope.

Electrical measurements were performed on the pellets with an Agilent B2911A unit. The surfaces of the pellets were polished, and silver electrodes were sputtered on each surface. The current–voltage measurements were carried out at room temperature to obtain the electrical conductivity.

Reflectivity spectra of the pellets (made from electrode powder) were measured under vacuum at room temperature using a home-made Fourier transform infrared microspectrometer (μFTIR) based on a Bruker IFS 66/v. The experiments covered the spectral range of 100–10 000 cm^−1^, which includes the far infrared (FIR), mid-infrared (MIR) and part of the near infrared (NIR) domains. The optical conductivity spectra were deduced from the reflectivity spectra using variational dielectric function analysis.^29^

To understand the influence of doping on the new electrode materials, conductivity measurements were realized using a 4-point probe setup consisting of four equally spaced tungsten metal tips with finite radii. Each tip was supported by springs on the other end to minimize sample damage during probing. A high impedance current source was used to supply current through the outer two probes; a voltmeter measured the voltage across the inner two probes to determine the sample resistivity. The typical probe spacing was set as 1 mm.

## 3. Results and discussion

### 3.1. XRD, TEM and SEM characterizations

**Fig. 1:**
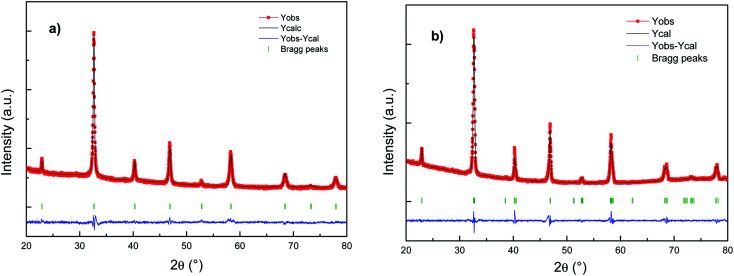
Rietveld fitting results for the X-ray patterns of (a) LSCF Cu_800 (cubic) and (b) LSCF Cu_1200 (rhombohedral) single-phase measured at room temperature.

Fig. 1 shows the refinement results (XRD patterns) of the as-prepared Cu doped LSCF powders sintered at 800 °C 1 h (LSCF Cu_800, Fig. 1a) or at 1200 °C 1 h (LSCF Cu_1200, Fig. 1b). For each figure, the intensities of the measured and calculated spectra are represented with dots and continuous lines, respectively. Under the spectra, the position of the Bragg reflections is indicated. The difference between the observed and the calculated profiles is plotted at the bottom.

The X-ray diffraction results indicate single phase cubic/rhombohedral structures with no superlattice peaks. LSCF Cu_800 corresponds to La_0.6_Sr_0.4_Co_0.2_Fe_0.8_O_3_ (PDF-04-018-5108) indexed in a cubic structure with the *Pm*3̄*m* space group with a cell parameter of around 3.8 Å. Meanwhile, LSCF Cu_1200 corresponds to La_0.6_Sr_0.4_Co_0.2_Fe_0.8_O_3_ (PDF-01-083-6426) indexed in a rhombohedral structure with the *R*3̄*c* space group with a cell parameter close to the more common hexagonal settings of *a*_h_ = *a*_p_√2, *c*_h_ = 2*a*_p_√3, *γ* = 120°. The rhombohedral structure results from distortions in the ABO_3_ structure due to slight deviations in the A and B-site cations and oxygen ion radii with increasing temperature.

Similar analyses were performed with the pure LSCF powder and the Nb and/or Cu doped LSCF powders. The results were compared with a commercial pure LSCF powder named LSCF HP.

Table 1 shows the sintering temperatures for each compound along with the lattice parameters and reliability factor *χ*^2^.

**Table 1:** Sintering temperatures for each compound along with their lattice parameters and reliability factors *χ*^2^

	Sintering temperature (°C) for cubic ©/rhombohedral ® phase	Lattice *a* (Å)	Lattice *c* (Å)	*χ* ^2^
LSCF	1000 ©	3.876(4)	—	2.44
LSCF Nb	1200 ©	3.913(2)	—	1.94
LSCF Cu_800	800 ©	3.877(0)	—	2.62
LSCF Cu_0.07_ + Nb_0.03_	900 ©	3.886(5)	—	2.59
LSCF Cu_0.05_ + Nb_0.05_	1000 ©	3.891(5)	—	1.99
LSCF HP	900 ©	3.888(2)		1.30
LSCF Cu_1200	1200 ®	5.494(3)	13.368(4)	3.47

The addition of niobium increases the cell volume and the sintering temperature compared to those of LSCF and LSCF Cu_800. The lattice parameter increases exponentially with the Nb rate for the doped LSCF samples. Niobium (rNb^5+^ = 64 pm) has a similar ionic radius to iron/cobalt (rFe^3+^/rCo^2+^: 55/65 pm) compared to copper (rCu^2+^ = 73 pm), which has a larger ionic radius. Instead, the increasing lattice parameter for the LSCF Nb can be explained by the fact that the Nb–O bonds lengthen while the Cu–O bonds shorten during charge transfer. Further investigations could offer a better understanding. Additionally, the sintering temperature (to achieve the cubic single phase) increased with Nb addition to the Cu-doped LSCF. One possible explanation is the higher fusion temperature for niobium compared to that for copper. Combining systematic electron diffraction (ED) and energy dispersive spectroscopy (EDS) analyses of numerous grains showed the good crystallinity and homogeneous cationic compositions of the samples. The chemical analysis of several crystallites by EDS is in agreement with the nominal compositions.

**Fig. 2:**
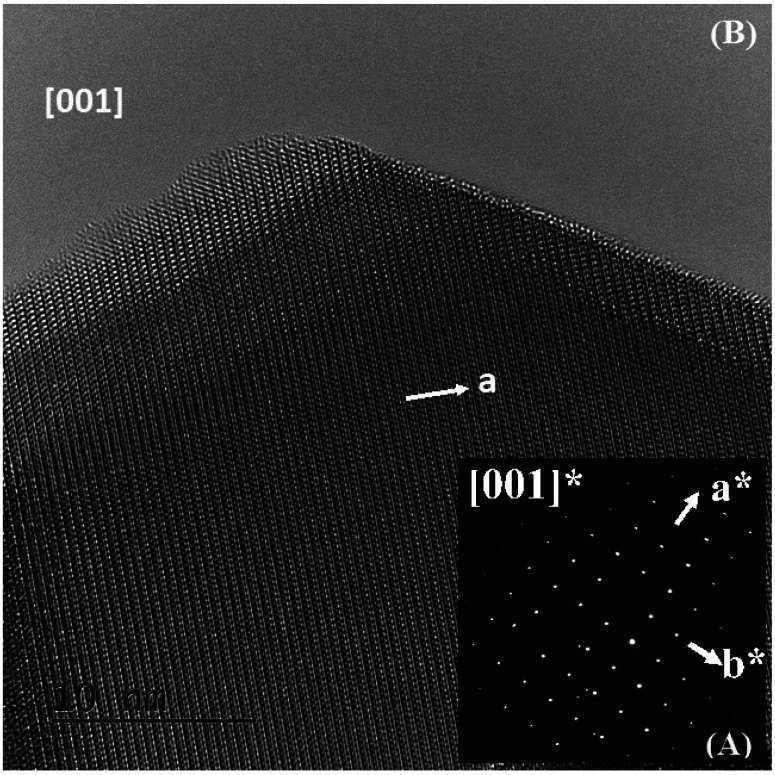
(A) ED pattern of the [001] zone axis and (B) the corresponding lattice image.

The ED patterns of all the crystallites are characteristic of the *Pm*3̄*m* – type average structure, with cell parameters close to *a*_p_ (*a*_p_ being the parameter of the cubic perovskite cell close to 3.8 Å). An example of the [001] ED pattern is presented in Fig. 2A, and the corresponding lattice image is shown in Fig. 2B. This image shows the regularity of the contrast, and no defects can be observed.

### 3.2. Infrared and transport measurements

**Fig. 3:**
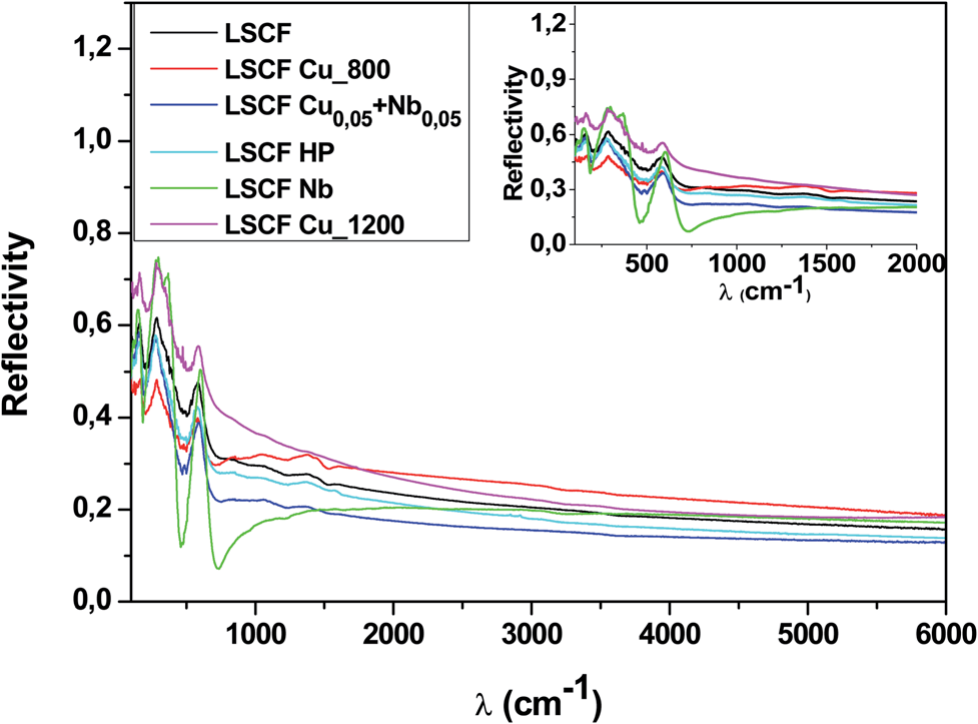
Reflectivity spectra at 300 K of pure and doped LSCF pellets between 100 and 6000 cm^−1^. The inset displays a zoom between 100 and 2000 cm^−1^.

Fig. 3 shows the room-temperature infrared reflectivity spectra for LSCF and doped LSCF pellets as a function of light wavenumber (*ω* = 1/*ν*, where *ν* is the light frequency).

The Nb reflectivity of LSCF has the typical shape of an insulator: sharp peaks attributed to phonon modes in the FIR (low energy, *ω* ≤ 800 cm^−1^), almost flat low reflectivity levels on average in the MIR and NIR (around 20% between 2000 and 6000 cm^−1^) and a type of “gap” between them (around 800 cm^−1^).

In contrast, LSCF Cu_1200 has the reflectivity of a “bad” metal. The overall reflectivity level decreases as *ω* increases, suggesting the presence of a Drude peak, the signature of free carriers. With respect to LSCF Nb, the phonon modes are notably screened; this is another hint of a “bad” metallic response, as in a “good” metal, these modes would be almost unnoticeable. The “gap” between the phonon modes and the MIR response is nonexistent. It is useful to note that LSCF Cu_1200 is the only sample that is not cubic but rhombohedral.

All the other samples fall between those two extreme cases, with some phonon mode screening, some “gap” filling and varying MIR responses between them. We can expect some conducting response from these samples; however, it will be lower than that of LSCF Cu_1200.

**Fig. 4:**
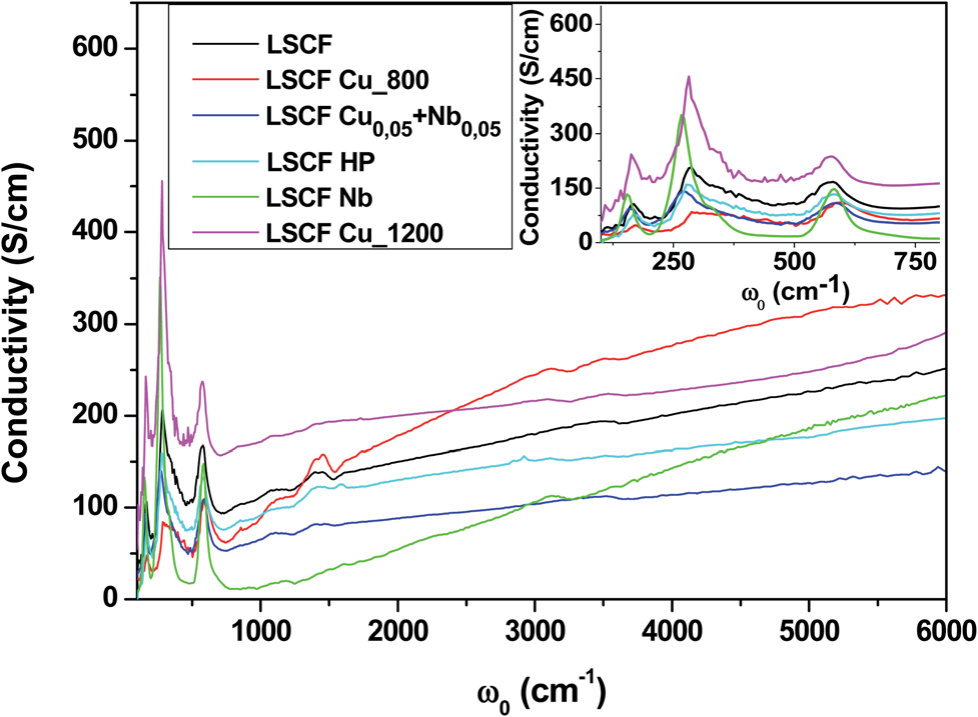
Optical conductivity spectra of pure and doped LSCF pellets at room temperature between 100 and 6000 cm^−1^. The inset displays a zoom between 100 and 800 cm^−1^.

To extract quantitative information from the reflectivity spectra and determine the optical conductivities, we used a Variational Dielectric Function method (VDF)^29^ (which is equivalent to a Kramers–Krönig transformation). The results are shown in Fig. 4. The three low-frequency peaks are the phonon modes, which correspond to the three IR-active modes of the cubic perovskite structure ABO_3_: from lower to higher energy, the external mode (the movement of cation A against the oxygen octahedral), the bending (of the B–O–B bond) mode and the stretching (of the B–O bond) mode.^30^ Any structural distortion would induce a higher number of modes: for example, 25 were observed for the orthorhombic structure and 8 for the rhombohedral structure. As we can see in the insert, cationic substitutions on the B site induced some phonon shifting. The adjustment of the optical conductivity using Drude–Lorentz oscillators allowed us to quantitatively extract the phonon mode positions (energy *ω*_0_) and widths (damping *G*). As we can see in the inset of Fig. 4, the bending mode is asymmetric for all samples, and to obtain a reasonable adjustment of this mode, the use of two oscillators (named bending 1 and bending 2) is necessary. The issue of structural distortion can already be raised. The adjustment parameters of the phonon modes are exposed in Table 2.

**Table 2:** Phonon positions (*ω*_0_) and widths (*G*) in cm^−1^ for the pure and doped LSCF samples

Sample	External mode		Bending mode 1		Bending mode 2		Stretching mode 3	
	*ω*_0_	*G*	*ω*_0_	*G*	*ω*_0_	*G*	*ω*_0_	*G*
LSCF	168.8	48.05	287.1	61.01	364.8	170.53	572.9	83.71
LSCF Cu_800	172.2	47.44	298	58.95	367.4	161.46	600.6	84.02
LSCF Nb	155.9	25.55	269.3	40.52	331.3	57.07	580.1	57.99
LSCF Cu_0.05_ + Nb_0.05_	161.3	40.46	271.3	71.72	351.1	136.48	582.0	79.89
LSCF HP	169.7	40.58	281.9	64.73	360.1	160.14	579.8	84.46
LSCF Cu_1200	168.2	74.53	286.7	66.85	382.2	171.41	571.4	61.89

The overall level of the conductivity spectra at low frequency is related to the metallicity of the sample; the higher the level, the more metallic the sample. However, proper analysis of the conductivity of the sample requires the zero-frequency extrapolation of the optical conductivity, which can be approximated by taking the lowest frequency value (100 cm^−1^) in the insert of Fig. 4. We retrieved the qualitative interpretation of the reflectivity: LSCF Cu_1200 is the most conductive sample, and LSCF Nb is insulating. Ignoring the phonon modes, the linear extrapolation at low frequency of the optical conductivity below 300 cm^−1^ allows us to extract the semi-conducting gap, which is around 0.1 eV (800 cm^−1^) for LSCF Nb and close to 0 eV for LSCF Cu_800. All the other samples have no gap and are bad metals. In Table 3, we report the lowest frequency (100 cm^−1^) optical conductivity to compare it to the transport measurements.

**Table 3:** Comparison of the conductivity values obtained from different methods

Sample	Conductivity (S cm^−1^)		
	Optical conductivity (at 100 cm^−1^)	Electrical conductivity_2 points	Electrical conductivity_4 points
LSCF	30	54	24
LSCF Cu_800	22	69	24
LSCF Nb	7	10	0.5
LSCF Cu_0.07_ + Nb_0.03_	—	47	14
LSCF Cu_0.05_ + Nb_0.05_	26	24	16
LSCF HP	7	5	4
LSCF Cu_1200	63	71	76

The conductivity values obtained from different methods are shown in Table 3.

The conductivity from the four-point probe method is smaller than that obtained from the two-point method, probably because of the lower contact and spreading resistance for the first method. However, the 4-point method is more suitable for measurements of small conductivity, such as electrode materials, because of the bad contact resistance. The tendency of the lowest value for LSCF Nb to the highest for LSCF Cu_1200 is similar for the two methods. The values go from the lowest value for LSCF Nb to the highest for LSCF Cu_1200. Therefore, niobium decreases the conductivity values, while copper increases them. The conductivity values for the mixed compositions of Cu + Nb are between these two limits. It is important to note that the values of electrical conductivity from the four-point probe method are similar to those for the optical conductivity at 100 cm^−1^. We can conclude that the 4-point measurement values are equivalent, within the limit of the errors, to the real conductivity values for electrode materials such as LSCF.

The electrical conductivity results from the 4-point method show the effects of the different dopings on the electrical character of the LSCF compounds. According to these results, Nb decreases the electrical properties to 1 S cm^−1^. This is close to that of commercial LSCF (LSCF HP), which has very low conductivity compared to the other samples. The best composition obtained concerning the electrical measurements is Cu-doped LSCF (LSCF Cu_800 and LSCF Cu_1200), with electrical conductivities of 24 and 76 S cm^−1^, respectively. As shown in Table 3, the Cu_0.05_ + Nb_0.05_ doped LSCF has a conductivity close to that of pure LSCF.

### 3.3. LSCF cubic phase stability on YSZ + GDC symmetrical cells

At high temperature, LSCF tends to change its crystallographic structure, moving from cubic to rhombohedral with increasing temperature. The material must maintain its cubic structure at the operating temperature of an SOC. To evaluate the structure stability, the cells were submitted to several heat treatments at 800 °C in stages of 24 h.

**Fig. 5:**
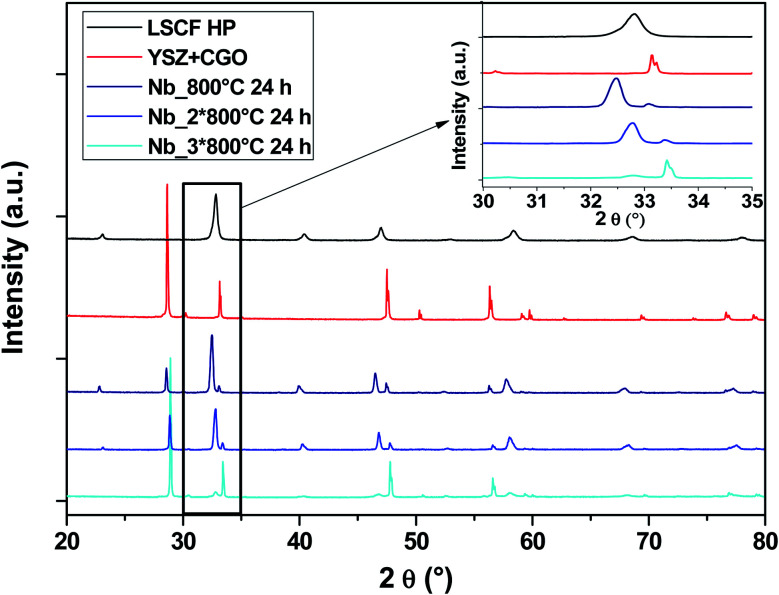
XRD patterns of cells after heat treatments for commercial LSCF and Nb-doped LSCF. The inset displays a zoom between 30 and 35°.

Fig. 5 shows the diagrams of the three Nb-doped LSCF cells after the heat treatments (blue diagrams). All the peaks are well identified either by the main cubic structure, observed with the commercial LSCF sample (black diagram), or by the phase linked to the support YSZ electrolyte and GDC protective layer (red diagram). There are no parasitic phases or structural changes, as can be seen in the zoom between the 2*θ* range of 30–35°.

**Fig. 6:**
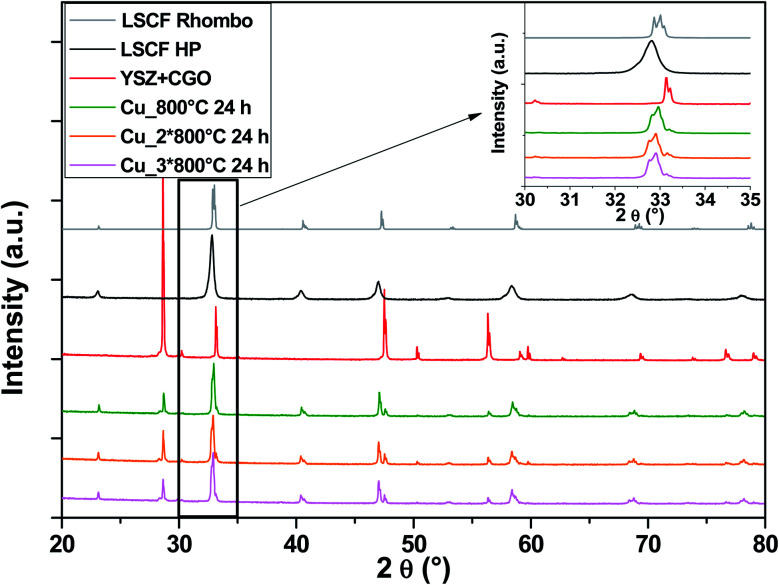
XRD patterns of cells after heat treatments for commercial LSCF and Cu-doped LSCF. The inset displays a zoom between 30 and 35°.

In the case of Cu-doped LSCF (Fig. 6), a structural change is observed: all peaks belong to the rhombohedral lattice system (grey diagram). This structural change was maintained after three heat treatments. Moreover, this phase is known for its stability at very high temperature. On the other hand, it was found that the cubic structure represents the best structure to ensure compatibility between the different components. Unlike Nb, it seems that Cu does not stabilize the cubic structure of LSCF.

Co-doping the LSCF composition (Nb + Cu) can be a solution to group together several characteristics: structural stability can be maintained while retaining good electrical properties. Co-doping Nb and Cu in an electrode structure has been already shown to be beneficial to preserve the cubic phase at room temperature and to efficiently improve the vacancy concentration in other materials, such as SrCoO_3−*δ*_.^31^

**Fig. 7:**
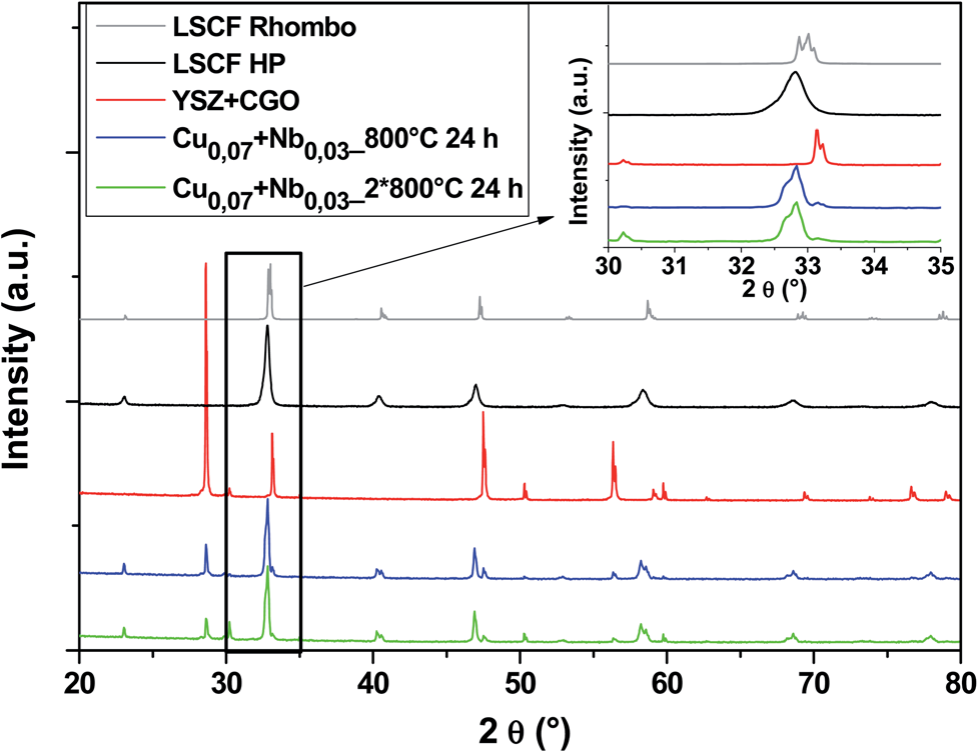
XRD patterns of cells after heat treatments for commercial LSCF and Cu_0.07_ + Nb_0.03_-doped LSCF. The inset displays a zoom between 30 and 35°.

Fig. 7 shows diagrams of the Cu_0.07_ + Nb_0.03_ doped LSCF cells after heat treatments (blue and green diagrams). This composition crystallized in the rhombohedral phase (grey diagram). We can therefore conclude that the amount of Nb is not enough to stabilize the cubic structure.

**Fig. 8:**
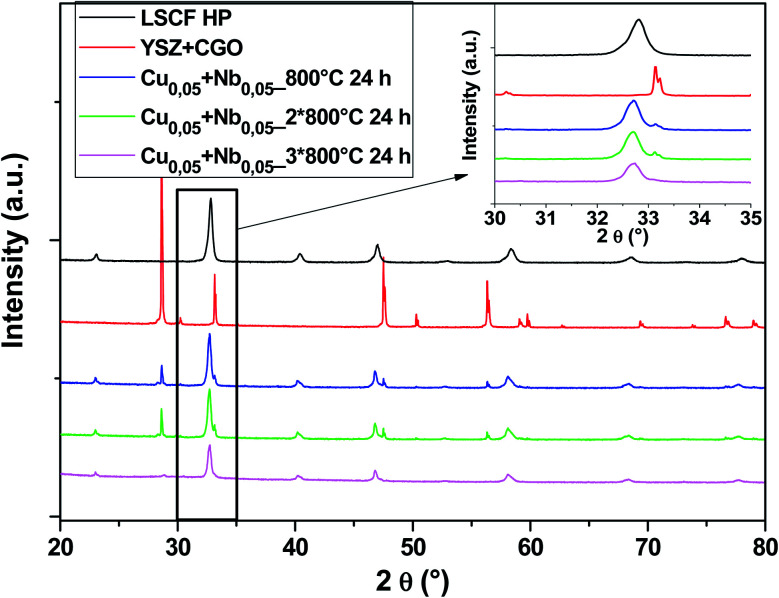
XRD patterns of cells after heat treatments for commercial LSCF and Cu_0.05_ + Nb_0.05_-doped LSCF. The inset displays a zoom between 30 and 35°.

Another composition with 50% Cu and 50% Nb in the B-sites was tested. The diagrams in Fig. 8 show that there are no parasitic phases. All peaks are identified either by the cubic structure of LSCF or by the substrate (YSZ + GDC symmetrical cell). LSCF Cu_0.05_ + Nb_0.05_ maintains its cubic structure even after several heat treatments (blue, green and pink diagrams).

**Fig. 9:**
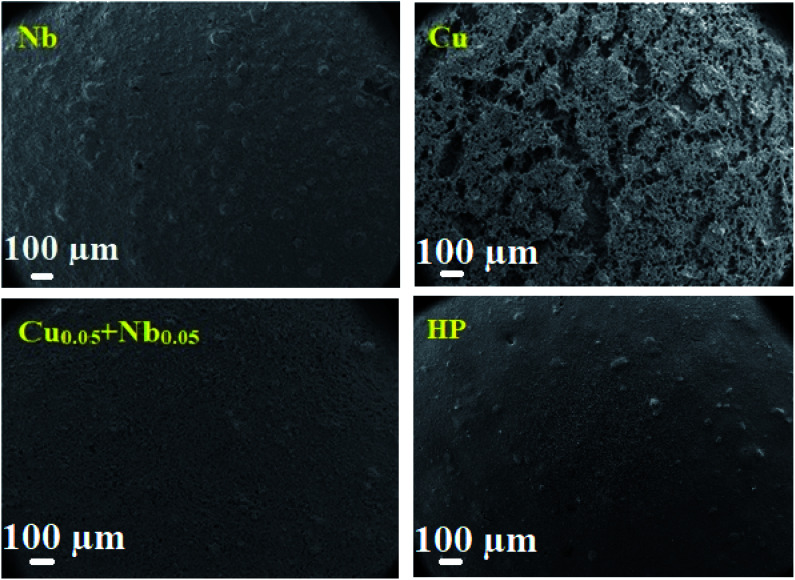
Surface SEM micrographs of the cells for the Nb, Cu, Cu_0.05_ + Nb_0.05_-doped LSCF and commercial LSCF (HP) after 3 heat treatments at 800 °C during 24 h.

Fig. 9 shows the SEM images of Cu, Nb and Cu_0.05_ + Nb_0.05_-doped LSCF after three heat treatments at 800 °C. The sample with Cu-doped LSCF appears to be very porous, with a discontinuous surface. However, a homogeneous shape is observed for the Nb and the Cu_0.05_ + Nb_0.05_-doped LSCF samples. The homogeneous structure is relatively similar to the one classically obtained with commercial LSCF (LSCF HP). This provides an additional reason to validate the Cu_0.05_ + Nb_0.05_-doped LSCF compound as a good electrode material.

### 3.4. Discussion

The reflectivity spectra in Fig. 3 and the conductivity spectra in Fig. 4 show that doping with a small amount of Cu or Nb does not greatly change the electrical properties. All the graphs have similar shapes of a conductor (such as a correlated metal) except for the Nb-doped LSCF sample, which is more insulating. The reflectivity spectra show drastic modifications in intensity below 2000 cm^−1^ when the dopant is changed but have similar trends at high frequency.

It seems that the metal–oxygen bonds could be modified during the redistribution of the charges between the 3d orbitals of copper or 4d orbitals of niobium with the 3d orbitals of iron/cobalt *via* the 2p orbital of oxygen.^31^ X-ray diffraction measurements have shown that the length of the Cu–O bonds shortens (decreasing the cell volume) during charge transfer, while the Nb–O bonds lengthen (increasing the cell volume).

Tahini *et al.*^32^ have shown that Nb ions have a strong bonding character in bulk SrNbO_3_; therefore, Nb is efficient in preserving oxygen stoichiometry, as it acts as an oxygen trap. This cation has also been found to be very effective in stabilizing the structure and improving the oxygen permeability of SrCoO_3_.^20^ On the other hand, the introduction of Nb has been found to reduce the electrical conductivity, mainly because increasing the number of non-conducting Nb–O bonds interrupts the carrier transport.^33^ This characteristic was observed for the Nb-doped LSCF compounds. In contrast, the introduction of Cu enhanced the conductivity, possibly because more Cu doping leads to the formation of more oxygen vacancies.^31^

**Fig. 10:**
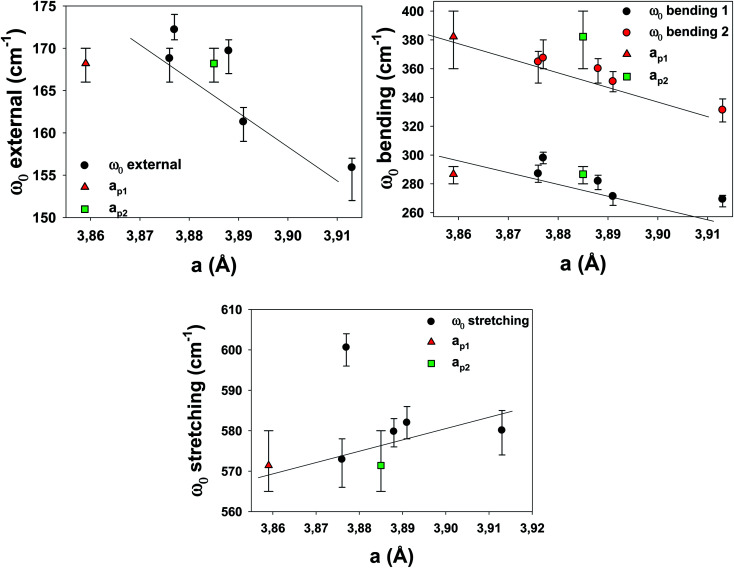
Phonon mode energy *ω*_0_ dependence on the lattice parameter a. For the rhombohedral sample LSCF Cu_1200, two lattice parameters are obtained: *a*_p1_ = *a*_h_/√2 (red triangle), and *a*_p2_ = *c*_h_/2√3(green square).

**Fig. 11:**
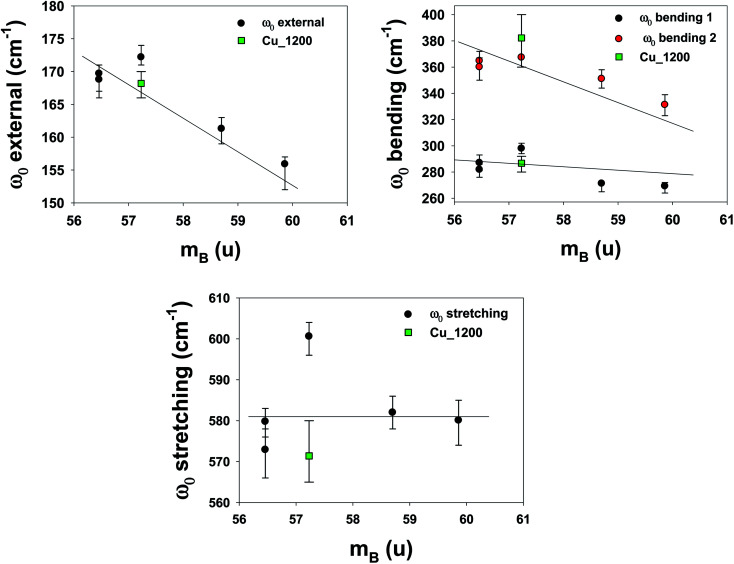
Phonon mode energy *ω*_0_ dependence on the average atomic mass of the B-site *m*_B_. For the rhombohedral sample LSCF Cu_1200, the atomic mass *m*_B_ is represented by the green square.

To understand the effects of B-site substitution in the lattice, we plot in Fig. 10 and 11 the phonon mode energy dependence *ω*_0_ with the lattice parameter *a* and with the average atomic mass of the B-site *m*_B_ (the latter calculated using the chemical formula of the compound), respectively, for the cubic samples. The rhombohedral Cu_1200 will be considered later.

As we can see, both the external and the bending modes exhibit a red shift with the increase of *a* and *m*_B_. However, the stretching mode shows a blue shift with the increase of *a* but no clear evolution with *m*_B_.

The red shifts are consistent with the usual mass effect (
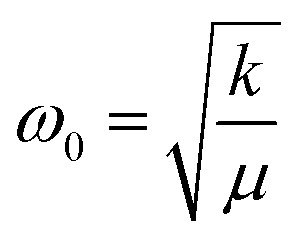
,^34^ where *k* is the spring constant and *μ* is the reduced mass), even if we would naively expect such an effect to be stronger for the modes directly involving the BO_3_ octahedra (the bending and the stretching modes), which is not the case.

Similarly, a red shift is generally observed when *a* increases, as the effective spring bond between atoms decreases when they move away from each other. This is the case for the external and bending modes, but not for the stretching modes. Therefore, the mode-Grüneisen coefficients^35^ are positive (as usual) for the external and bending modes and negative for the stretching mode. Negative mode-Grüneisen coefficients have also been observed in other perovskite compounds.^36^

As we can see in Table 2, the widths *G* of the peaks are ≈50% larger with respect to LSCF Nb, except for the bending 2 modes, which are ≈150% larger. The mode widths are generally related to structural defects, high temperature or unresolved modes (which are the signatures of decreased symmetry). The notably smaller widths for all modes in LSCF Nb are compatible with the fact that the Nb stabilizes the structure better because it introduces fewer defects.

Overall, the complex dependence of the phonon mode energies with *a* and *m*_B_ as well as the larger width of the bending 2 mode and the technical difficulties of the DRX refinement procedure for the cubic samples strongly suggest that they are distorted cubic: a structural deformation of the BO_3_ octahedra could be involved with B-site substitution.^30^ The number of Drude–Lorentz oscillators used in the adjustment procedure of the optical conductivity, which was already increased from 3 (the number of IR active modes for the cubic perovskite) to 4, could be increased even more, as the large bending 2 damping values (for all samples except LSCF Nb) point to more unresolved modes, which strengthens the hypothesis of symmetry lowering.

Finally, the phonons of the rhombohedral Cu_1200 were added to the plots (Fig. 10 and 11). Because there are two different lattice parameters *a* and *c*, two different pseudo-cubic parameter values *a* were deduced (3.859 and 3.885 Å). The external and bending 1 modes of Cu_1200 seem to better follow the general trend when the *a* = 3.885 Å value is chosen, whereas the bending 2 mode better fits the general picture with *a* = 3.859 Å. The stretching mode is compatible with both pseudo-cubic lattice parameters. This could be explained if we consider that the structural deformations induced by the symmetry lowering involve different octahedra along the *c*-axis and the (*ab*)-plane.

Further, *ab initio* calculations could offer a better understanding of the electronic and structural properties of these materials, and especially their behavior (stability of the structure) in long-term heat treatment of the complete cell. The substitution at the B sites may also be understood as a chemical pressure effect.

## 4. Conclusions

The La_*x*_Sr_1−*x*_Co_*y*_Fe_1−*y*_O_3_ compound (LSCF) is one of the most studied materials in the literature as an oxygen electrode for SOCs. The performance of this “mixed” conductor degrades due to Sr segregation and migration at the electrolyte/electrode interface, which causes instability of the structure and formation of secondary phase.

Doping with various atoms, such as Nb, Cu and Cu + Nb, in then B-site (Co/Fe) was elaborated. The pure and doped LSCF samples were deposited by screen-printing on symmetrical cells (YSZ electrolyte covered by a 4 μm thick GDC barrier layer on both faces) to be tested.

Nb was found to stabilize the LSCF structure in the cell even after three heat treatments. The best electrical conductivity at room temperature was found for the Cu-doped LSCF compound at around 70 S cm^−1^.

Combining the structure stability and properties, we show by XRD, SEM, infrared and transport measurements that the composition LSCF Cu_0.05_ + Nb_0.05_ best meets the required criteria for a very promising oxygen electrode for IT SOCs. It maintains the stable cubic structure after heat treatment and has better conductivity compared to a standard LSCF (LSCF HP). The deposits with this powder are homogeneous and nanometric, and they maintain the stable cubic single-phase structure of the electrode.

## Conflicts of interest

There are no conflicts to declare.

## Supplementary Material
